# Aromatase Inhibitor-Induced Erythrocytosis in a Patient Undergoing Hormonal Treatment for Breast Cancer

**DOI:** 10.1155/2015/784783

**Published:** 2015-06-02

**Authors:** Sri Lakshmi Hyndavi Yeruva, Stanley Madu Nwabudike, Onyekachi Henry Ogbonna, Patricia Oneal

**Affiliations:** Division of Hematology and Oncology, Howard University Hospital, 2041 Georgia Avenue, NW, Washington, DC 20060, USA

## Abstract

Aromatase inhibitors (AIs) are most commonly used for breast cancer patients with hormone receptor positive disease. Although the side effect profile of aromatase inhibitors is well known, including common side effects like arthralgia, bone pain, arthritis, hot flashes, and more serious problems like osteoporosis, we present a case of an uncommon side effect of these medications. We report the case of a postmenopausal woman on adjuvant hormonal therapy with anastrozole after completing definitive therapy for stage IIIB estrogen receptor-positive breast cancer, who was referred to hematology service for evaluation of persistent erythrocytosis. Primary and known secondary causes of polycythemia were ruled out. On further evaluation, we found that her erythrocytosis began after initiation of anastrozole and resolved after it was discontinued. We discuss the pathophysiology of aromatase inhibitor-induced erythrocytosis and reference of similar cases reported in the literature.

## 1. Introduction

An estimated 234,190 patients will be diagnosed with invasive breast cancer in 2015 and 40,730 patients will die from the disease this year, as per the American Cancer Society [1]. Of these patients, those that have estrogen receptor-positive tumors receive hormonal therapy designed to suppress the tumor by reducing estrogen levels. In postmenopausal women with estrogen receptor-positive breast cancer, the hormonal therapy of choice is an aromatase inhibitor whose mechanism of action ultimately causes a reduction in estrogen production. The most common side effects of aromatase inhibitors are related to their antiestrogen effect and are widely known. We, however, present a case of aromatase inhibitor-induced erythrocytosis, an uncommon side effect of aromatase inhibitor use.

## 2. Case Report

We report a case of a 57-year-old woman who developed erythrocytosis while on anastrozole for estrogen receptor-positive breast cancer. Our patient has a history of hypertension and invasive poorly differentiated ductal carcinoma of the right breast, clinical stage T4N0M0 (IIIB), estrogen receptor-positive and progesterone receptor-positive, and human epidermal growth factor receptor-negative. Her breast cancer had been treated initially with neoadjuvant chemotherapy (Adriamycin and cyclophosphamide) followed by lumpectomy with positive margins and then subsequent bilateral simple mastectomy with reconstruction. She then completed adjuvant chemotherapy with paclitaxel and was started on hormonal therapy with anastrozole subsequently. On routine follow-up MRI scans, she was discovered to have breast implant rupture and was scheduled for implant replacement. However, on preoperative workup, she was found to have erythrocytosis and, thus, was referred to our hematology clinic in January 2015 for evaluation and management.

At the time of consultation, she reported feeling well except for intermittent headaches and difficulty sleeping. She denied neurologic, cardiovascular, and respiratory symptoms. She had no erythromelalgia or constitutional symptoms. She also had no evidence of bleeding diathesis or recent infections. She smoked crack cocaine about twice per week and also endorsed drinking about 3 to 12 ounces of beers daily. She had a 2-pack-year smoking history but quit 1 year prior to consultation. She endorsed using marijuana.

Her medications included hydrochlorothiazide, lisinopril, and anastrozole. Physical examination was unremarkable. Particularly, she had no hepatosplenomegaly, hirsutism, or elevated blood pressure. Laboratory studies at this point revealed the persistence of erythrocytosis, with hemoglobin of 16.8 g/dL and hematocrit of 51.3%, white blood cell count of 5,500/mm^3^, and platelet count of 189,000/mm^3^. Complete metabolic panel was normal. Serum erythropoietin level was 3.4 (reference: 2.6–18.5). We requested additional work-up to exclude a myeloproliferative process: we obtained a JAK2 mutation analysis with reflex to exon 12 testing to rule out polycythemia vera. Fluorescent In Situ Hybridization (FISH) analysis of BCR-ABL translocation was ordered to rule out chronic myeloid leukemia. Both tests were negative, hence ruling out a myeloproliferative process.

Having ruled out myeloproliferative disorders, we decided to look for possible secondary causes of polycythemia and requested chest X-ray, pulmonary function tests, and echocardiography. These, also, were unremarkable. There was no suggestion of chronic lung disease or structural heart disease. On close review of her laboratory data and medication history, however, we noted that her polycythemia started in September 2014; around the same time, patient was started on anastrozole. Further laboratory investigation showed elevated serum total testosterone of 84 ng/dL (7–40 ng/dL), free testosterone of 2.4 (0–9.5 ng/mL), and DHEA sulfate of 253 *μ*g/dL (29.4–220.5 *μ*g/dL). With a possible diagnosis of secondary polycythemia due to medication, she was asked to discontinue anastrozole for one month.

On return to clinic for followup in February 2015, four weeks after anastrozole was discontinued, repeat hemoglobin and hematocrit were 13.8 g/dL and 40.6%, respectively, serum total testosterone was 50 ng/dL, free testosterone was 1.2 ng/dL, and DHEA sulfate was 170 *μ*g/dL. Upon discussion with her oncologist, her hormonal therapy was switched to tamoxifen and her hemoglobin remained in normal range.

## 3. Discussion

Aromatase inhibitors (AIs) are recommended for adjuvant hormonal therapy in postmenopausal women with hormone receptor-positive breast cancer. There are two types of aromatase inhibitors: nonsteroidal inhibitors such as anastrozole and letrozole and irreversible steroidal inhibitors such as exemestane. The most common side effects seen are related to deficiency of estrogen and include increased risk of bone loss and fractures, arthralgia and bone pain, hypercholesterolemia, vaginal dryness and atrophy, dyspareunia with decreased libido, hot flashes, night sweat, and heat intolerance [2]. These adverse effects mirror those seen in menopause and perimenopause due to estrogen deficiency. Other less common side effects include nausea, diarrhea, rash, hair thinning, headache, neurologic effects, and visual disturbance [2, 3].

Though erythrocytosis is not a major side effect reported with aromatase inhibitor use, aromatase inhibitors prevent peripheral conversion of testosterone to estradiol (and androstenedione to estrone), leading to increased levels of testosterone, which would explain the erythrocytosis seen in our case. Androgens have been known to have a stimulatory role in red blood cell production, which is one of the reasons why men have a higher red cell count. This effect of androgens on erythrocytosis is also seen at higher altitudes as reported by Gonzales et al. [4, 5]. The complete mechanisms by which testosterone induces erythrocytosis are still largely unknown. Recent investigations have shown a direct relationship between testosterone and increased erythropoietin (EPO) levels. This increase in erythropoietin is thought to be one of the mechanisms of testosterone-induced erythrocytosis. In a study by Bachman et al., increased EPO was seen in the first 3 months of administration of testosterone [6]. Ip et al. demonstrated that higher trough serum levels of testosterone, rather than duration of treatment, were shown to be predictive for the development of polycythemia in hypogonadal men receiving testosterone replacement therapy [7]. This supports the theory of testosterone-induced erythrocytosis and, by extension, aromatase inhibitor-induced erythrocytosis.

Another proposed mechanism of testosterone-induced erythrocytosis is by the suppression of hepcidin levels [6, 8]. Hepcidin is a liver derived peptide, which is an important regulator of iron homeostasis. Low hepcidin is associated with increased iron absorption, increased systemic iron transport, and iron bioavailability for erythropoiesis [6, 8, 9]. Testosterone also upregulates the expression of genes involved in erythrocytosis such as GATA-1, FOG-1, and other GATA-dependent genes [6]. This could increase erythropoietin sensitivity and stimulate erythropoiesis. It is noteworthy that these studies about the hematologic effects of testosterone were in hypogonadal men receiving exogenous androgens.

In our case, we noted a significantly elevated serum total and free testosterone level in our postmenopausal patient with erythrocytosis, which was abnormal. Erythropoietin level, however, was normal. Regardless, we held her aromatase inhibitor for a month and noted a drastic decline in both her testosterone level and erythrocyte counts upon repeated testing. This shows the association between testosterone and erythrocytosis and reveals one of the pathophysiologic mechanisms behind aromatase inhibitor-induced erythrocytosis.

On review of the medical literature, to the best of our knowledge, only three cases of polycythemia following the administration of aromatase inhibitors have been reported. The first case was of two boys treated with letrozole for hypogonadism who subsequently developed erythrocytosis. [10]. The second case involved a 79-year-old lady with localized hormone positive breast cancer who was treated with exemestane and developed erythrocytosis. She had initially been treated with letrozole, but that was discontinued secondary to nausea according to the authors of that report. The patient had required phlebotomies while she was undergoing hematologic evaluation for erythrocytosis, prior to discontinuation of the aromatase inhibitor [11].

Although aromatase inhibitors are used frequently in postmenopausal breast cancer patients, erythrocytosis seems to be an uncommon side effect as most of these patients have anemia either due to the cancer itself or due to treatment administered. Our patient was treated with anastrozole, a different aromatase inhibitor compared to what is described in earlier reported cases associated with increased erythrocyte counts. As noted in prior case reports, erythrocytosis has been noted in patients on both steroidal and nonsteroidal aromatase inhibitors and is, therefore, not peculiar to a particular class.

## 4. Conclusion

As erythrocytosis in our patient resolved within one month after discontinuation of anastrozole without need for aggressive invasive intervention, we hypothesize that the mechanism of aromatase inhibitor-induced erythrocytosis involved a physiologic increase in testosterone as seen with exogenous testosterone administration [12]. Oncologists, therefore, need to be mindful of the possibility of erythrocytosis as a side effect of aromatase inhibitors and consider discontinuing the drug before subjecting patients to more invasive procedures like phlebotomies. Since not many patients on aromatase inhibitors develop erythrocytosis, determining which patients are susceptible to this effect is subject for further research.
